# Associations between neighborhood environment and sense of community belonging in urban China: Examining mediation effects of neighborly interactions and community satisfaction

**DOI:** 10.3389/fpubh.2022.1105473

**Published:** 2023-01-12

**Authors:** Yang Du, Huaxiong Jiang, Zhuo Huang, Haoran Yang

**Affiliations:** ^1^Faculty of Science, College of Urban and Environmental Sciences, Peking University, Beijing, China; ^2^Faculty of Geographical Science, Beijing Normal University, Beijing, China; ^3^The Center for Modern Chinese City Studies, East China Normal University, Shanghai, China; ^4^School of Urban and Regional Science, East China Normal University, Shanghai, China

**Keywords:** neighborhood design, walkability, community belonging, structural equation modeling (SEM), urban China

## Abstract

**Introduction:**

Improving sense of community belonging (SCB) would help people live longer, happier lives. Although the importance of neighborhood environment on SCB is stressed in the literature, few studies have paid attention to perceived environment, as well as consider mediation effects such as neighborhood social interactions and place satisfaction.

**Methods:**

Relied on a sample of 1051 respondents in Shanghai in 2018, this study investigates the associations between both objective and perceived neighborhood environment attributes and SCB in urban China, mediated by neighborly interactions and community satisfaction using structural equation modeling.

**Results:**

The results suggest that the influence of perceived neighborhood environment on SCB is more prominent than that of objective neighborhood environment. In detail, perceived pedestrian facilities and perceived leisure facilities are vital to SCB, while among objective neighborhood environmental elements, the influence of land use entropy, park density and street greenery are significant. Then, neighborhood environmental attributes can influence SCB by affecting neighborly interactions and community satisfaction. We also identify gender differences in the effects of neighborhood environment upon SCB.

**Discussion:**

Given increasing awareness of the connection, neighborhood environment may prove to be valuable assets to improve individuals' psychosocial constructs such as SCB.

## 1. Introduction

Community plays a vital role in shaping our physical and mental health (1–4). It offers stability, solidity, and secureness that facilitate individuals' prosperity and growth. Francis et al. (3) state that people often own high levels of intellectual wellness and positive emotions when they live in communities that are inclusive, stable, and safe, and acceptance and respect across differences, including the vulnerable groups. Due to huge impacts, we recently see increasing research and urban planning interest in the relationship between the built environment and social and mental constructs like sense of community belonging (SCB) (5, 6).

According to McMillan and Chavis (7), SCB is conceptualized as a perception and feeling that community members perceive important to each other. It is a belief or conviction that their physical and social needs and demands would be satisfied through the promise to be together. French et al. (4) point out that SCB embodies the common, mutual and social values that are owned by community members, and represents the adherence, attachment, and coherence that can be embedded in and integrated into geographic establishments. In urban planning and health studies, SCB is closely related to many neighborhood-based results and consequences such as membership, neighborhood attachment, fulfillment of needs, engagement, etc. (8). It is argued that although increase in global mobility homogenizes sense of place, causing a loss of identity and traditional local community, people emotionally tend to search for local propinquity, belonging and identity (4, 5). Thus, many scholars urge that policy makers, researchers and urban planners alike create the opportunities and conditions that facilitate and enhance SCB in living neighborhoods (9–11).

In the field of urban planning, one of the centric goals of New Urbanism principles is to enhance SCB and improve people's sense of belonging (12). The underlying assumption is that the patterns the street grid is organized, and the structure a neighborhood is planned would influence neighborly interactions and activities and influence people's sense of that place (13). For instance, neighborhoods and blocks providing a mix of shops, offices, recreation, and residences would make walking more convenient, services more cost-effective and living places more comfortable and enjoyable (4). Then, communities designed to promote face-to-face interactions and develop social associations and support systems offer pedestrian-friendly streets and blocks, working and housing in close proximity, and approachable public spaces, and accommodate multimodal transportation such as transport use, walking, cycling and driving (1). Consequently, members of these neighborhoods experience greater residential satisfaction and belonging.

Although a range of studies examined the relationship between the built environment and SCB, evidence is mixed, and there is a lack of studies discussing the specific characteristics of the subjectively measured neighborhood environment that contribute to SCB and under what conditions (4, 11, 14). In fact, perceived neighborhood attributes and elements that increase opportunities for neighborly interactions and communication may be a stronger predictor of neighborhood satisfaction and SCB (11). For instance, people have a higher level of SCB when perceiving the circumstance to be secure, having the chance engaging community activities, and owning local enjoyable spots and sites within the community (15).

Then, there is an ignorance by researchers of the gender differences in the association between neighborhood environment and SCB. As Lo (16) suggests, psychosocial constructs like SCB vary between males and females since their use of neighborhood space and perceptions of residential environment differ. For instance, it is often pointed out that females would encounter more social and environmental restraints and the physical-social environment seem to have greater influences on their daily behavior than males (17). Despite the importance, the gender issue relevant to the relationship between neighborhood environment and SCB is hardly explored.

Furthermore, previous research examining the pathways from environmental elements to SCB often overlooks the mediation effects of neighborly interactions and place-based satisfaction like community satisfaction (6, 11, 18). According to Smith (19), the neighborhood environment may influence SCB *via* its impacts on neighborly social interactions and place satisfaction.

To fill the gaps, the aim of this paper is to investigate how objective and subjectively measured neighborhood environment attributes influence SCB, mediated by neighborly interactions and community satisfaction. Based on data from Shanghai in 2018, we use structural equations modeling to analyze structural relationships. This study has the following three key contributions. First, it enriches the literature on the relationship between SCB and both objective and subjectively measured environmental elements in neighborhoods. Second, this study assesses the relative importance of perceived environmental attributes in predicting SCB. Third, this study reveals new pathways from the environment to SCB, that is, neighborly interactions and community satisfaction play critical mediation roles in the structural relationship.

## 2. Literature review

According to McMillan and Chavis (7), a strong SCB is often closely related to place satisfaction, physical and mental health, and life wellbeing, whereas a low SCB can result in feelings of alienation, insignificance, loneliness, and social and cultural isolation. Thus, taking action to facilitating a greater connection of residents to their community would improve their feelings of belonging, trust, and security, and thereafter their physical and mental health (7, 14).

Among various public policies to improve SCB, built environment and neighborhood planning is one of many possibilities and ways (4, 15). In the literature, it is commonly recognized that the neighborhood environment is a contributor to SCB as it influences individuals' emotion, feeling and satisfaction with many of their daily life aspects (6). A range of studies have discussed the association between neighborhood environmental attributes and SCB (4, 5, 20). For instance, some authors found that communities with low and medium floor area ratios would give members of community more private space and offer more available and approachable community resources, which brings more positive feelings and sense of belonging to their communities (21, 22). On the contrary, Foster et al. (23) found that communities instability attributes such as large number of floating population and high crime rates would reduce people's chances of establishing relationships and building dialogues with one another, and consequently reducing people's perception of community. Note that the relationship between the built environment and SCB is underestimated (4, 15). Because of the widely differed prediction techniques, it shows that some studies reported a positive association between neighborhood environment elements and SCB, whereas others show negative effects. The results are hybrid and thus more empirical studies are needed to understand the associations.

Then, previous studies disproportionately center on the association of objective neighborhoods environment on SCB (24), whereas the effects of the subjectively measured neighborhood environment have been largely ignored, except for a few exceptions (4, 11). According to Guo et al. (11), subjectively measured neighborhood environments may contribute to SCB given that people would feel a greater connection to their community when they have a better perception and feeling of their living circumstances. The perceived environment consists of residential characteristics relevant to the social exchanges and interplay among community members, which are vital for enhancing community cohesion and integrity (3). A number of studies show that people's satisfaction and attitudes toward their communities is closely related to perceived convenience of local transport, perceptions about whether services are accessible easily, perceived greenery or attractiveness, perception of incivilities, and perceptions of violent and property crimes (6, 7, 25, 26).

In fact, perceptions of neighborhood attributes play as much of a role in influencing individuals' sense of their surroundings as objective features, and sometimes even more vital (15). In their study of walkable cities, Hoehner et al. (27) identified that perceived and objective environmental measures would influence people's levels of physical activity and their attitude toward their local communities. Rather than the objectively measured environment, the perceived environmental measures have larger effects and was necessary for improving individual's feeling, understanding, perception and attitude (12, 28). In another study, Ries et al. (29) found that perceived plentifulness of facilities were connected with increased community physical-social activity such as neighborly interactions and communication. Abdullah et al. (9) also identified that only perceived availability of parks and sports facilities had a significant influence on residential satisfaction rather than objective elements. It shows that perception could be an unignorable mediator between the objective built environment and SCB. Thus, examining such intervening factors is vital to develop and implement effective strategies and policies to promote sense of community and belonging.

Consistent with certain previous studies, the gender differences in SCB seemed to vary significantly (30, 31), indicating that while some neighborhood environments might influence the SCB of men and women, the influences differ considerably. It is said that females are more likely to use the neighborhood space for their recreation and leisure activities compared to males (32). Consequently, residential environment shows a considerable variation in predicting SCB between females and males. For instance, studies indicate that females seem to be more sensitive to environmental features (30, 32). The finding agrees with increasing proofs that there exist stronger environmental relationships in women regarding their use, perceptions and sense of their community. It implies that support of physical and psychosocial environment is critically needed to facilitate females to use neighborhood space and could be a strategy for reducing the gender inequality. However, most neighborhood-based studies merely offer the overall relationship between neighborhood environment and neighborhood satisfaction and SCB, ignoring the likelihood that this relationship differs between males and females (33). In other words, the overall effect potentially conceals vital information and message concerning how neighborhood environment has a differed influence on the SCB of females and males. Thereafter, more attention is needed to discern gender differences in the impacts of environmental attributes on SCB.

In addition, the pathways from the environment to SCB hardly consider the mediation effects of mediators like satisfaction domains and neighborly social interactions. First, recent studies indicate that place-based satisfaction would influence perception of community and their life satisfaction (18). When people perceive walkable street quality, high accessibility, quality architecture and design, and environmental quietness (26), they are more likely to be satisfied with their neighborhoods and have better sense of their community. Second, neighborly interactions could play a mediating role in the association of the neighborhood environment with SCB (4). In a pedestrian-friendly and walkable environment, an intensified use of public spaces improves the frequency of information sharing, exchange and interactions between members of community, thus facilitating the establishment of social ties and affiliation among neighbors (6). According to Jorgensen et al. (34), neighborly interactions promote neighboring relations and improve individuals' perception of empowerment from others. For instance, in a survey concerning neighborhood interactions, Smith (19) states that 86.3% of respondents revealed that they often stop and talk with community members and 98% would help others in an emergent situation. The results demonstrated that neighborly social interaction enable residents to experience instrumental and psychosocial support, consequently leading to high residential satisfaction, SCB, and sense of belonging (35).

Despite the argued potential of the mediation effects, studies have less focused on the potential pathways from neighborly interactions and community satisfaction to SCB. Individuals engaged in the community experience faithful psychological and social help, neighborhood stability and growth, few social isolation and community satisfaction, all of which contributes to the improvement of SCB (15). Therefore, it is necessary to distinguish compound associations among the environment, neighborly interactions/community satisfaction, and SCB.

## 3. Methodology

### 3.1. Data collection

In this study, we use two types of data to analyze the association of neighborhood environment with SCB. One is the Shanghai Built Environment and Resident Behavior Survey data, collected by the East China Normal University, China. The data set includes respondents' sociodemographic data, perception data incorporating individuals' perception of neighborhood environment, and feeling and attitude toward their residential community. Two is the objective neighborhood environment data, which was calculated based on the respondents' location address provided in the Built Environment and Resident Behavior Survey.

**Figure 1:**
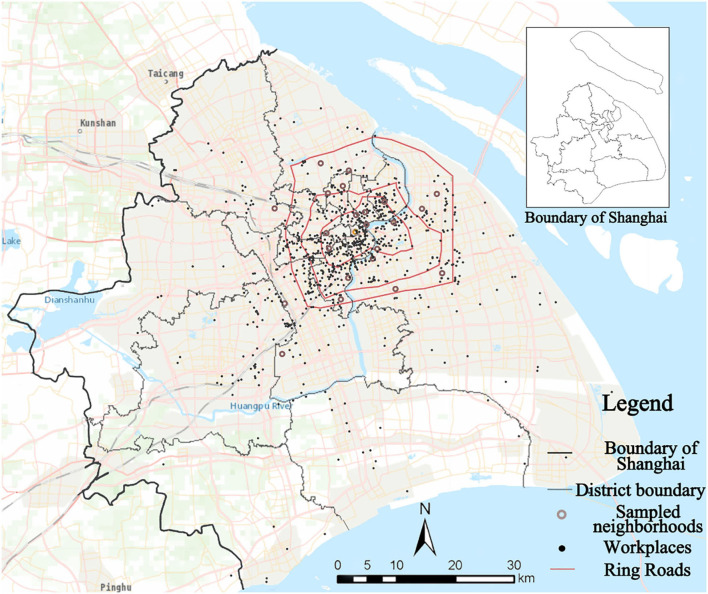
Distribution of the sampled neighborhoods (36).

The survey was implemented in urban Shanghai, from August 2018 to February 2019. By adopting a stratified sampling method, respondents were randomly selected from 38 housing estates of 30 primary sampling units—jie dao or town in the 13 districts of Shanghai (Figure 1). In each housing estate, 35 households were invited to participate the questionnaire survey (the targets include either the household head or spouse between the ages of 18 and 60 years old). The survey collected a total of 1,127 respondents and 1,051 respondents finished all the questions, with a response rate of 93.3%. Respondents' living locations was geocoded for further analysis of the built environment characteristics.

### 3.2. Conceptual framework and variables

**Figure 2:**
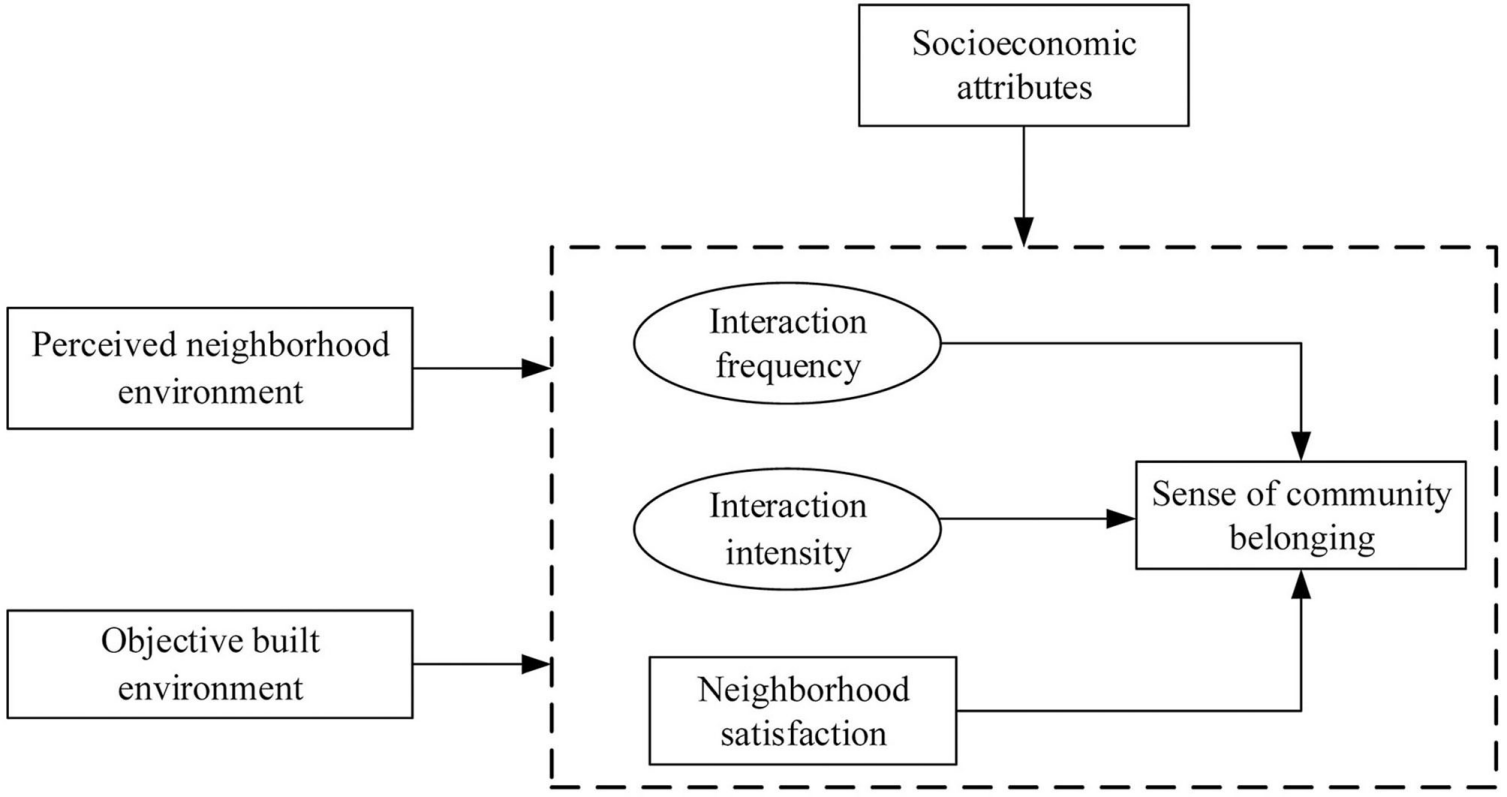
Conceptual framework.

Based on the literature review, a conceptual framework is developed to examine how objective and perceived neighborhood environment influence SCB, mediated by neighborly interactions and community satisfaction (Figure 2). We hypothesize that individual perception of the environment as well as objective environment influence SCB through the mediation effects of neighborly interactions/neighborhood satisfaction. Demographic and socioeconomic attributes are also hypothesized to influence individuals' SCB directly.

SCB is the dependent variable. Normally, a single and overall question is deemed of being good reliability, thus the question “To what extent you have a feeling of community belonging?” was formulated to assess the domain satisfaction. Since we hypothesize that neighborly interaction mediates the association between the built environment and SCB, neighborly interaction is established as a latent variable, comprised of interaction frequency and interaction intensity. Interaction frequency includes 4 four-point Likert-type scale items (1 = extremely low, 5 = extremely high), and the mean score for the 4 items was measured and adopted for each participant. Interaction intensity was measured by three five-point Likert-type scale items; and questions interpreting the variable include “I am willing to help other members of community,” “I have a good relationship with other members of community,” and “I think other members of community are trustable”. Then, the question “How satisfied are you with your residential neighborhood?” was used to measure the mediating variable neighborhood satisfaction, designed with a 5-point scale, ranging from “1” (very dissatisfied) to “5” (very satisfied).

Based on respondents' geolocation, we measured the objective built environment attributes in residential area through creating a 1,000 m circular buffer. Street view data based on Baidu Maps was employed to measure individuals' perception of the built environment on the ground. The street network of Open Street Map for 2018 was applied to generate regular sampling points every 30 m along the streets. We obtained the street view images with a dimension of 1,024× 1,024 pixels for each sampling point. The deep machine learning model was applied to divide the amount of greenery per image. The aggregated greenery level of each sampling point within each residential neighborhood was calculated through using the average greenery of the four images.

Wood et al. (15) point out that leisurely walking environmental attributes influence SCB. Thus, some walkability-related measures were also applied. Floor area ratio was obtained by dividing a building's total floor area to the area of the circular buffer. The number of parks within residential neighborhoods were measured within the 1,000 m buffer. By employing the Shannon entropy index, we measured the land use diversity relied on 15 different Point of Interest (POI) categories (e.g., utilities, and retail and wholesale). We also used the Gaode Map platform to calculate the number of road intersections within the 1,000 m buffer to operationalize the street connectivity.

Perceived neighborhood environmental attributes were obtained from the questionnaire survey. Based on the work by Nguyen et al. (37) and Wood et al. (15), variables depicting subjectively measured environment attributes include public transport convenience, pedestrian and leisure facility, safety, road accessibility, and environmental quality. Note that these variables were also developed on a five-point Likert scale, ranging from “1” (extremely unimportant) to “5” (extremely important).

Sociodemographic variables were used as control variables, which include gender, age, hukou, marital status, education level, income, perceived overall health, car ownership, children under 18, household structure, and living duration (years).

### 3.3. Descriptive analysis

The descriptive analysis of the variables is provided in Table 1. Among the 1,051 respondents, 570 were males (54.2%) and 481 were females (45.8%), with a mean age value of 39.56. Concerning the education level, 65.2% of the respondents have received a college degree or above. Percentages of respondents with low-, middle- and high-level income account for 31.7, 34.4, and 33.9% respectively. Then, most of the respondents have local hukou (76.9%), and the average living duration is around 12.15 years. More than 75% (76.7 %) of the respondents are married and 45.7% of them have children under the age of 18. The percentage of car ownership is around 45%.

**Table 1:** Explanations of variables and descriptive statistics (*N* = 1,051).

**Variables**	**Description**	**Explained**	**Distribution**	
**Endogenous variables**			**Mean**	**SD**
Sense of community belonging	To what extent you have a feeling of community belonging (1 = extremely low, 5 = extremely high)	Categorical variable: {1, 2,…,5}	3.94	0.729
Interaction frequency	Please evaluate the frequency you interact with your neighbors in the following aspects:		2.18	0.861
Frequency of dialogue	(1 = never, 4 = often)	Categorical variable: {1, 2,…,4}	2.88	0.897
Frequency of visit	(1 = never, 4 = often)	Categorical variable: {1, 2,…,4}	1.88	0.850
Frequency of going out together	(1 = never, 4 = often)	Categorical variable: {1, 2,…,4}	1.99	0.893
Frequency of helping each other	(1 = never, 4 = often)	Categorical variable: {1, 2,…,4}	1.99	0.806
**Interaction intensity**				
Mutual aid	I am willing to help other members of community (1 = very disagree, 5 = very agree)	Categorical variable: {1, 2,…,5}	3.92	0.688
Inter-relationship	I have a good relationship with other members of community (1 = very disagree, 5 = very agree)	Categorical variable: {1, 2,…,5}	4.01	0.645
Trust	I think other members of community are trustable (1 = very disagree, 5 = very agree)	Categorical variable: {1, 2,…,5}	3.88	0.69
Neighborhood satisfaction	How satisfied are you with your residential neighborhood (1 = very dissatisfied, 5 = very satisfied)	Categorical variable: {1, 2,…,5}	3.99	0.599
**Exogenous variables** **Objective built environment**				
Road intersection	Number of road intersections within 1,000 m	Continuous variable: R+	33.51	18.463
Park density	Number of parks within 1,000 m	Continuous variable: R+	2.64	2.284
Population density	Population density within 1,000 m	Continuous variable: R+	27,225.17	17,674.226
Land use entropy	Land use entropy within 1,000 m	Continuous variable: R+	2.29	0.148
Street greenery	Street greenery within 1,000 m	Continuous variable: R+	0.20	0.064
Floor density	Floor area ratio within 1,000 m	Continuous variable: R+	1.56	0.657
Perceived neighborhood environment			
Perceived public transport convenience	To what extent do you think public transport in your surrounding is convenient?	Categorical variable: {1, 2,…,5}	4.46	0.558
Perceived pedestrian facilities	To what extent do you think pedestrian facilities in your surrounding are sufficient?	Categorical variable: {1, 2,…,5}	4.19	0.639
Perceived leisure facilities	To what extent do you think leisure facilities in your surrounding are sufficient?	Categorical variable: {1, 2,…,5}	4.15	0.667
Perceived neighborhood safety	To what extent do you think your neighborhood is safe?	Categorical variable: {1, 2,…,5}	4.74	0.502
Perceived road accessibility	To what extent do you think the road systems in your surrounding is accessible?	Categorical variable: {1, 2,…,5}	4.40	0.623
Perceived environmental quietness	To what extent do you think your living environment is quiet?	Categorical variable: {1, 2,…,5}	4.49	0.605
Perceived air quality	To what extent do you think the air quality in your surrounding is satisfactory?	Categorical variable: {1, 2,…,5}	4.62	0.557
**Socioeconomic attributes**			**Frequency**	**%**
Gender	Gender (%) (Female = 1)	Categorical variable: {0, 1}		
	0		570	54.2%
	1		481	45.8%
Age	Age (years)	Continuous variable: R+	Mean	SD
			39.56	10.171
Education	Education level	Categorical variable: {1, 2,…,4}	Frequency	%
	Middle school or below		123	11.7%
	High school		243	23.1%
	College		665	63.3%
	Master or above		20	1.90%
Hukou	Hukou (%) (Yes = 1)	Categorical variable: {0, 1}		
	0		243	23.1%
	1		808	76.9%
Living duration	Living duration (years)	Continuous variable: R+	Mean	SD
			12.45	9.932
Income	Income	Continuous variable: R+	Frequency	%
	Low (< 100,000 CNY)		333	31.7%
	Mid (100,000–200,000 CNY)		362	34.4%
	High (>200,000 CNY)		356	33.9%
Children under 18	Children under 18 (yes = 1)	Continuous variable: R+		
	0		571	54.3%
	1		480	45.7%
Marital	Marital (yes = 1)	Categorical variable: {0, 1}		
	0		245	23.3%
	1		806	76.7%
Car ownership	Car ownership (yes = 1)	Categorical variable: {0, 1}		
	0		578	55.0%
	1		473	45.0%

Regarding objective neighborhood environment attributes, the average population density reaches 27 thousand people per square kilometer, while the average floor area ratio is 1.56. The mean value of land use entropy and the number of road intersections are 2.29 and 33.51, respectively. Street greenery and park density have an average value of 2.64 and 0.20 separately. In terms of the perceived environment, the average score of the perceived neighborhood safety is the highest (mean = 4.74), followed by perceived environmental air quality (mean = 4.62), perceived environmental quietness (mean = 4.49), perceived convenience of public transport (mean = 4.49), and perceived road accessibility (mean = 4.40). Perceived pedestrian facilities and perceived leisure facilities received relatively lower scores of 4.19 and 4.15, respectively. All indicators describing the latent variable interaction frequency have scores of <3 whereas that describing interaction intensity score larger than 3.8. In addition, the average value of neighborhood satisfaction is 3.99. SCB have a mean value of 3.94, indicating the relatively high level of SCB of the locals.

### 3.4. Method

This study employs structural equation models (SEMs) to investigate the relationship between built environment and SCB. Composed of measurement equation and structural equation, SEM is able to estimate both the causal relationships among observed variables and the influences of latent variables on others (38, 39). Besides, it can also reveal the nature of the mediation effect on the association of an independent variable with a dependent variable. A structural equation model with latent variables is generally composed of two equations (39, 40). First is the measurement equation which specifies the relationship between factor indicators (*F*_*kin*_) and the latent variable:


$$F_{kin}=\alpha_{ikn}z_{in}+\xi_{ikn}$$

where *F*_*kin*_ = factor indicator *k* of latent variable *i* for individual *n*, *z*_*in*_ = latent variable *i* for individual *n*, α_*ikn*_= coefficient for latent variable *i* and factor indicator *k*, ξ_*ikn*_ = measurement error term.

Second is the structural equation which specifies the relation of a predictor (either endogenous or exogenous) with other latent or observed variables:


$$Y_{n}=\beta_{i}z_{in}+\gamma_{j}X_{jn}+\varepsilon_{n}$$

where *Y*_*n*_ = dependent variable, either endogenous or exogenous, *X*_*jn*_ = observed variable *j* for individual *n*, β_*i*_ *and γ*_*j*_= coefficient values, ε_*n*_ = structural error term.

In this study, SCB was used as the final endogenous variable in the model, and interaction frequency, interaction intensity and neighborhood satisfaction as the mediating variables. Objective neighborhood environment, perceived neighborhood environment and socio-demographic variables are the exogenous variables. After confirmatory factor analysis (CFA) in the software AMOS26, the latent variables including interaction frequency and interaction intensity were constructed with an acceptable fit and internal consistency (Table 2). The VIF values of explanatory variables are below 5, indicating that no multicollinearity exists.

**Table 2:** Confirmatory factor analysis results.

	**Estimate**	**S.E**.	**C.R**.	***P***
Interaction intensity → Inter-relationship	1.000			
Interaction intensity → Mutual aid	0.941	0.035	27.236	***
Interaction intensity → Trust	0.923	0.035	26.458	***
Interaction frequency → Frequency of visit	1.000			
Interaction frequency → Frequency of chat	0.852	0.048	17.796	***
Interaction frequency → Frequency of outdoor activities	0.925	0.051	18.269	***
Interaction frequency → *Fr*equency of mutual aid	0.735	0.045	16.267	***

(1) The estimated correlation between interaction intensity and interaction frequency is 0.604 (>0.5).

(2)^***^P < 0.001.

Model fit c^2^/df. 2.719, CFI 0.995, AGFI 0.980, RMSEA 0.040, chi 24.472, df 9.

## 4. Results

### 4.1. Modeling fitness

We used the maximum likelihood (ML) method of estimation in this study. Note that the validity of ML theoretically depends on whether the SEM meets the assumption of multivariate normality of its variables. Therefore, we used a bootstrapping approach to draw repeated sample from the data (41, 42) and generate a sample of 5,000. Meanwhile, the bias-corrected bootstrap confidence intervals were used to detect significant effects.

All models fit the data adequately (Table 3). The χ^2^/*d*. *f* is 2.543 (values of 3 or less indicate a good fit), and other goodness-of-fit indices, such as RMSEA = 0.037 (values <0.05 indicate a good fit) and CFI = 0.949 (values range from 0 to 1, and values >0.9 are acceptable), also indicate that the model fit is good. Regarding the multilevel structural equation models, the fit indices also show an acceptable fitness.

**Table 3:** Goodness-of-fit statistics of the model and reference value.

	**χ^2^/d.f**.	**RMSEA**	**GFI**	**CFI**
Model 1. Overall model	2.543	0.038	0.955	0.949
Model 2. Multilevel SEMs	1.819	0.028	0.940	0.950
Reference value	< 3	< 0.05	>0.9	>0.9

### 4.2. Modeling results

#### 4.2.1. Relationship between the built environment and sense of community

Consistent with previous research, both objective and perceived neighborhood environment attributes have significant influences on SCB, but the influence of perceived attributes is more prominent (12, 28) (Table 4). In detail, perceived pedestrian facility has a positive and significant impact on the SCB. The result supports the finding by Lund (43) that in a pedestrian-friendly environment, people are more likely to use public spaces for information exchange and social activities, thus promoting a closer community relationship. Perceived safety has a positive impact on the SCB whereas perceived neighborhood quietness has a negative impact.

**Table 4:** Relationships between the neighborhood environment and SCB, mediated by neighborly interactions/community satisfaction.

**Variables**	**Interaction frequency**	**Interaction intensity**	**Community satisfaction**	**SCB**		
				**Total effects**	**Direct**	**Indirect**
**Endogenous variables**						
Interaction frequency	-	–	–	0.071	0.071	–
Interaction intensity	-	–	–	0.167^***^	0.167^***^	–
Neighborhood satisfaction	-	–	–	0.236^***^	0.236^***^	–
**Exogenous variables** **Perceived neighborhood environment**						
Perceived air quality	0.000	0.058	−0.019	0.023	0.018	0.005
Perceived public transport convenience	−0.120^***^	0.036	0.03	0.037	0.032	0.004
Perceived environmental quietness	−0.122^***^	−0.039	−0.011	−0.052	−0.034	−0.018
Perceived leisure facilities	0.158^***^	0.118^***^	0.068	0.032	−0.015	0.047^***^
Perceived pedestrian facilities	0.049	0.109^**^	0.125^***^	0.115^***^	0.064^**^	0.051^***^
Perceived road accessibility	0.031	−0.04	−0.003	−0.036	−0.031	−0.005
Perceived neighborhood safety	0.021	0.088^**^	−0.006	0.049	0.034	0.015
**Objective built environment**						
Floor area ratio	−0.071	−0.035	−0.02	−0.025	−0.009	−0.016
Land use entropy	0.005	0.046	0.031	0.086^*^	0.071^*^	0.015
Park density	−0.03	−0.041	0.042	0.076^**^	0.076^**^	0.001
Population density	−0.039	−0.003	−0.01	−0.012	−0.006	−0.006
Road intersections	0.009	0.051	−0.058	−0.028	−0.023	−0.005
Street greenery	−0.003	0.009	−0.03	−0.055^*^	−0.049	−0.006

^*^P < 0.1;

^**^P < 0.05;

^***^P < 0.01;

Model fit c^2^/d.f. 2.448, CFI 0.959, GFI 0.965, RMSEA 0.037, chi 528.737, df 216.

As for objective neighborhood environment, park density has a significantly positive effect on the SCB, indicating that the more parks there are around a community, the stronger the SCB belonging among the locals. Land use entropy has a positive impact on community sense, indicating that a mixed land use brings more positive feelings and sense of belonging to their residents. The influence of street greenery is negative, which contradicts with previous studies that street greenery positively influences SCB. One possible explanation is that when street greenery is relatively high, the benefits or utility of physical activities turns to be saturated, consequently resulting in low neighborhood satisfaction (44). Other variables like floor area ratio and population density have negative impacts on SCB belonging, which accords with previous studies (21).

**Figure 3:**
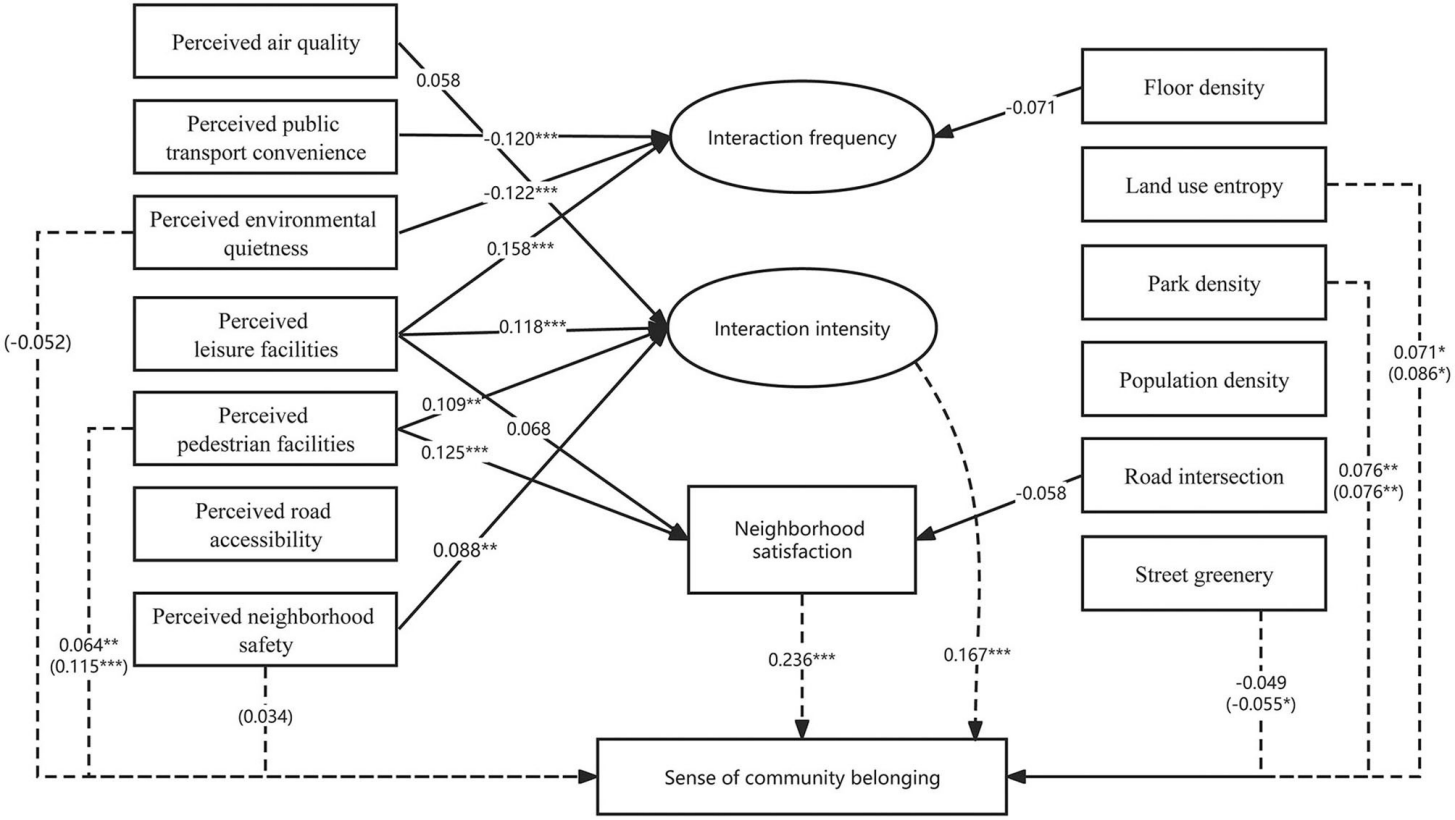
SEM analysis of the relationship between the built environment and SCB. (1) All the paths shown are significant at the 0.20 level (*P* < 0.20). (2) The dashed line shows the direct effect of variables to SCB, and values in parentheses represent total effects (the absence of a value before the parentheses means that the variable has no direct effect on SCB at the 0.20 level). Solid lines show the effect of exogenous variables on interaction behavior and community satisfaction. (3) ^*^0.05 ≤ *P* < 0.1; ^**^0.01 ≤ *P* < 0.05; ^***^P < 0.01.

When we consider about the influence path, the results show that neighborhood environment influences SCB by affecting neighborly interaction and neighborhood satisfaction (Figure 3). In other words, neighborly interaction and neighborhood satisfaction play a mediating role between subjective neighborhood environment and SCB.

First, interaction intensity and neighborhood satisfaction have partially mediating benefits between perceived pedestrian facility and SCB. The path coefficients of perceived pedestrian facility → interaction intensity/community satisfaction → SCB are all significant, indicating a significant mediating effect. Besides, perceived pedestrian facility also exerts a significant direct effect on SCB, consequently contributing a total positive effect on SCB. Then, perceived leisure facility is positively and significantly related with SCB *via* affecting neighborly interaction. Perceived leisure facility has a positively significant effect on both interaction frequency and interaction intensity, and the latter of which is positively and significantly related to SCB.

Perceived public transport convenience has positive effects on SCB, which means that the more convenient the perceived public transport, the lower the frequency of community interaction. A possible explanation of this odd result is that when public transport is perceived to be convenient, people tend to do more social activities outside their own community. Nevertheless, the overall influence of perceived convenience of public transport on SCB is still positive considering its positive impact on interaction intensity and community satisfaction, as well as its direct positive correlation with SCB.

Perceived neighborhood safety has a significantly positive effect on SCB by positively affecting interaction intensity. It is understandable that safer spaces would lead to a higher level of neighborly interactions, consequently better SCB. Perceived neighborhood quietness has a negative impact on SCB *via* negatively influencing interaction frequency. Note that this result differs from previous studies. One explanation is that the quieter a neighborhood is perceived, the less vibrant it is likely to be, thus leading to a lower level of SCB.

#### 4.2.2. Heterogenous impacts of the built environment on SCB between males and females

**Figure 4:**
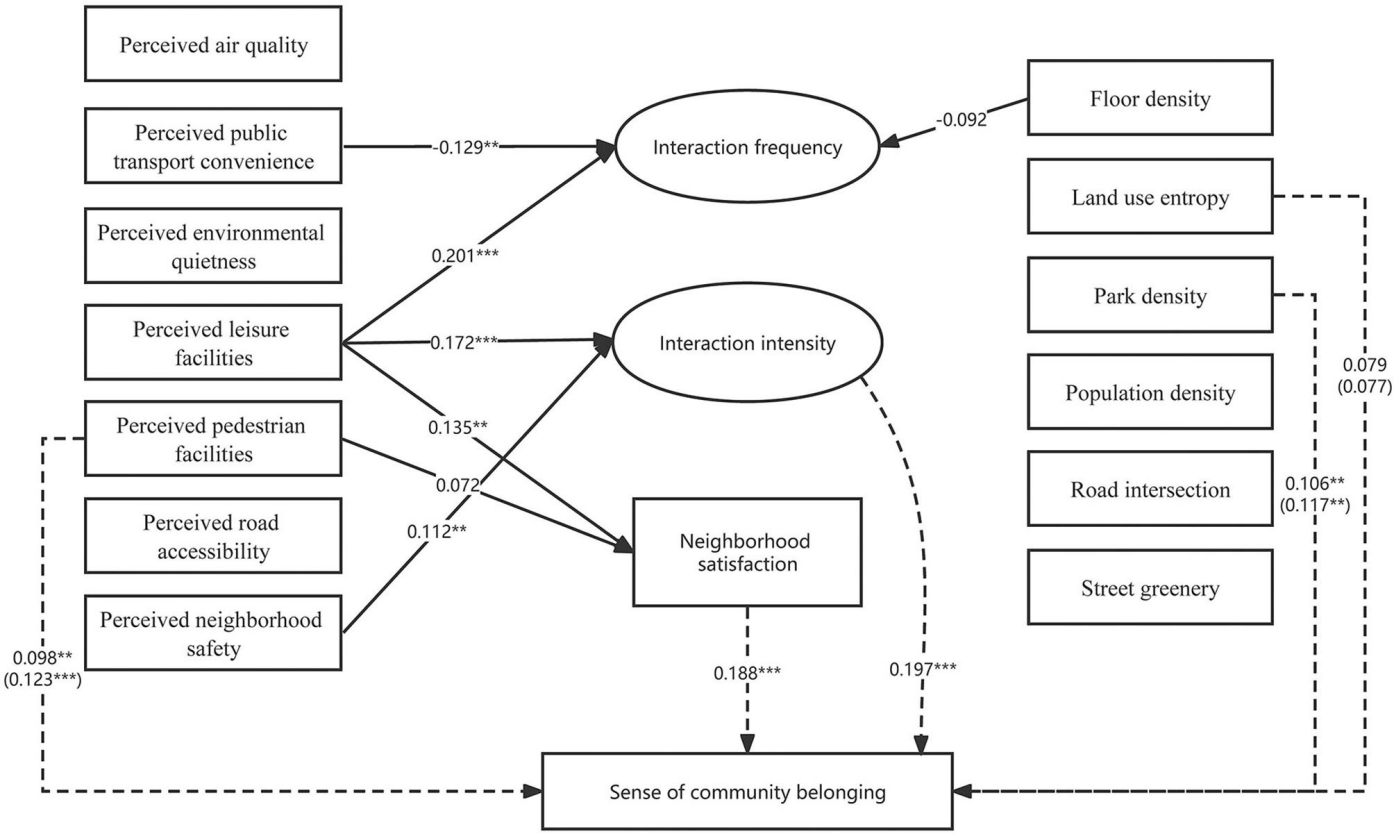
SEM analysis of the relationship between the built environment and SCB (female) (***p-value < 0.01, **p-value < 0.05, *p-value < 0.1).

**Figure 5:**
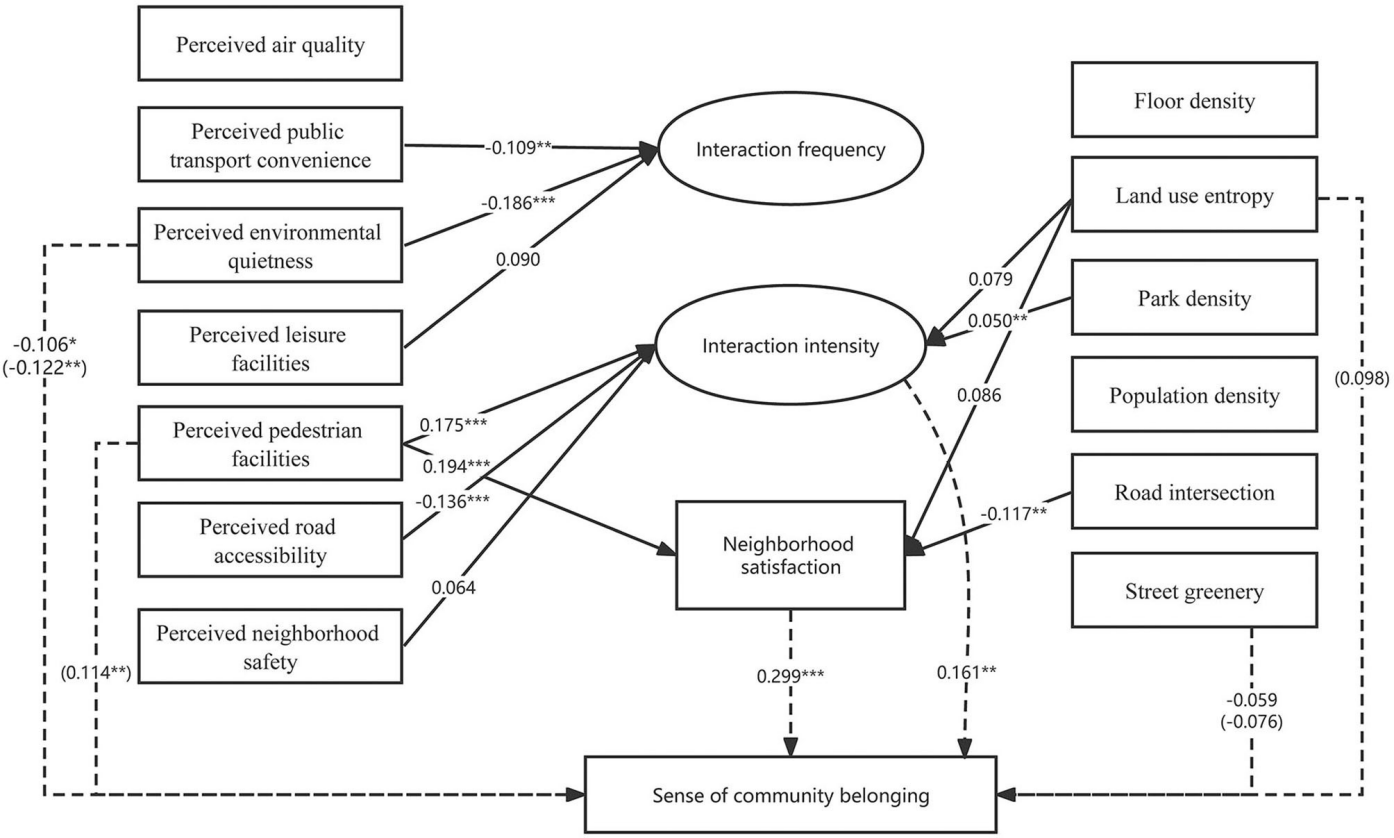
SEM analysis of the relationship between the built environment and SCB (male) (***p-value < 0.01, **p-value < 0.05, *p-value < 0.1).

The influence of neighborhood environment on SCB is different between males and females (Figures 4, 5). It shows that perceived neighborhood environment mainly has a significant influence on females' SCB, whereas both objective and perceived neighborhood environment attributes are significantly associated with males' SCB, and both of which are partially mediated by neighborly interaction and communication satisfaction. In the female group, those significant perceived neighborhood environment attributes include perceived leisure facility, perceived pedestrian facility, and perceived safety. In the male group, significant objective variables consist of park density and road intersections while those significant perceived variables comprise perceived pedestrian facility and perceived road accessibility.

Another finding is that although both females and males are sensitive to pedestrian or leisure facilities, females are more influenced by perceived neighborhood safety whereas males are more subject to the influence of road conditions like road intersections and road accessibility. In detail, the influence of perceived leisure facilities on SCB is significant in both the female and male group. However, road intersections and perceived road accessibility mainly significantly influence males' SCB, mediated by interaction intensity and community satisfaction respectively. In contrast, perceived neighborhood safety significantly influences SCB *via* influencing interaction intensity in the female group. This finding resonates with recent studies that there is evidence of substantial differences in perceptions of the importance of neighborhood environment between males and females (32).

## 5. Discussions

Researchers point out that the way neighborhood architectures and environments are designed shapes individuals' SCB, consequently individuals' mental health. The present study employs some indicators assessing SCB relevant to neighborly social interactions and satisfaction; and investigate its relationship with design characteristics of neighborhoods. However, research has seldom discerned the impacts of both objective and perceived neighborhood environment, as well as consider the mediation effects of neighborly interactions and community satisfaction. Therefore, efforts are made in this study to investigate the mechanism through which the relationships take place.

First, the significant effects of neighborhood environment confirm the importance of taking into account both objective and perceived measures of neighborhood environment in predicting SCB. Specifically, we found that both objectively measured and perceived neighborhood environmental elements are significantly associated with SCB. Our findings regarding the role of objective neighborhood environment in SCB, corroborates the principles of New Urbanism that high quality architecture, walkable pedestrian streets and urban design enhance SCB through invoking neighborhood social interactions (12). In this study, we identified that the number of parks, land use entropy, and street greenery are significantly associated with SCB. The finding resonates with previous studies that SCB tends to be higher in these neighborhoods that encourage more active travel like walking and to be featured with mixed land use, connected street networks and plenty of leisure facilities (45, 46).

Note that the importance of perceptions of neighborhood environment on SCB are also verified and confirmed. In this paper, individual's perceptions of the neighborhood's features and appearance of dwelling significantly influence interactions within the neighborhood and ultimately SCB. According to Talen (47), although objective environmental elements could encourage neighborly interactions among members of community, improvement to SCB may never occur. This is because simply considering connection between objectively measured environments and SCB ignores the complexity of the process how individuals perceive, understand, and decide (19). As Smith (19) states, feelings of affect and normative beliefs are positively related to a preference. Therefore, identifying and interpreting the interaction between individuals and environment is as important as measuring the objective environment itself. In this study, of particular relevance to SCB was the strong significant impacts of factors such as perceived public transport convenience, perceived neighborhood quietness, perceived leisure facility, and perceived pedestrian facilities. The finding resonates with some recent studies that perceived environmental elements in residential neighborhoods have a vital role in shaping social interactions or psychosocial constructs such as SCB, neighborhood cohesion, social support, and community connectedness. From this perspective, considering the influences of perceived environmental attributes on neighborhood dynamics is indispensable.

Second, the mediation effects of neighborly interactions and community satisfaction are verified and confirmed in this study, indicating the chained pathways between neighborhood environmental elements and SCB. According to French et al. (4), SCB is a stable assessment since individuals and citizens interpret and perceive their lives from long-term experiences and involvement within their surroundings. It is a combination of feeling and psychological SCB with multiple psychosocial domains (e.g., neighborly interactions, place-based satisfaction, social support and social capital) (7). Bottini (10) states that neighborly social interactions lighten ones' mood, reduce the risk of dementia and facilitate a sense of belonging and safety. It enables individuals to build positive psychosocial constructs with others such as social exchanges, civic engagement, friendliness, and trust; and ultimately helps improve people neighborhood sense and belonging (23). In this paper, frequency and intensity of neighborly interactions mediate the influence of perceived neighborhood environmental attributes such as perceived environment quietness and perceived leisure facility on SCB, especially on the female group. The findings confirm the argued importance of neighborly interactions in shaping individuals' feeling and psychological SCB. Thus, neighborly interactions become an important anchor of SCB.

Then, community satisfaction is also confirmed to be part of the chained pathways between neighborhood environmental elements and SCB. Hasanzadeh (48) state that residential neighborhoods have become the key spaces for people's daily activities. Members of community usually have high level of neighborhood satisfaction if they are satisfied with their living space that can fulfill their daily want, demand, requirement, and demands (49). In this paper, neighborhood satisfaction mediates the influence of perceived pedestrian facility on SCB. The identified new pathways from the environment to SCB enriches the literature on the relationship between SCB and perceived environmental elements.

Third, we also found heterogeneous influences of neighborhood environment on SCB between males and females; and the finding has implications for planning interventions based on gender difference. Firstly, it shows that females' SCB is mainly influenced by perceived neighborhood environment whereas males' SCB by both objective and perceived neighborhood environment attributes. This means males face more environmental restraints and experience greater opportunities for influence. The result differs a lot from previous studies that females faces more environmental restraints. According to Ma (50), although males deem that environmental features have a low influence on daily activities and emotional feelings, they still place considerable importance on them. In contrast, females often neglect the influence of neighborhood environmental features although they consider these features to be influential. Secondly, we identify that females' SCB is more influenced by perceived neighborhood safety whereas males' SCB more by road conditions like road intersections and perceived road accessibility. According to Jiang et al. (51), males spend more times on daily commuting, thus they put more weight on neighborhood environment like road conditions. As for females, a recent study by Hoffman et al. (52) shows that “women's perceptions of neighborhood sexual violence predicted perceived safety in their neighborhood”, consequently, they are more sensitive to neighborhood safety. The heterogenous influence between males and females is vital from the perspective of neighborhood planning and social justice. It suggests males and females experience differed environmental restraints, and thus differentiated pro-environmental actions and behavior among urban designers and planners help develop higher level of SCB.

Two key limitations are acknowledged. First, this paper presumes a linear relationship between neighborhood environment and SCB. However, increasing research criticize the restrained assumption through proposing alternative non-linear associations (36, 53). For instance, when street greenery is not too high, people tend to walk in their residential area and have high neighborhood satisfaction; but after street greenery becomes too high, the benefits or utility of walking turns to be saturated, consequently resulting in low neighborhood satisfaction (44). Thus, there is considerable need for researchers to focus on non-linear relationship between neighborhood characteristics and SCB. Second, the generalizability of the findings may be limited since Shanghai is chiefly featured with high urban density development. For instance, self-reported measures of the neighborhood characteristics may differ considerably across residents (18), indicating differences in behavioral preferences, which could interpret the result discrepancy across cities and countries.

## 6. Conclusion

There is an increasing interest in the association between neighborhood environment and estimates of psychosocial wellbeing like SCB. However, limited research has been conducted to examine how this relationship occurs. This study investigates the mechanism through which objective and subjectively measured neighborhood environment attributes influence SCB, with a special focus on the mediation effect of neighborly interactions and community satisfaction. The results suggest that the influence of perceived neighborhood environment on SCB is more prominent than that of objective neighborhood environment. In detail, perceived pedestrian facility and perceived leisure facility are vital to SCB, while among objective neighborhood environmental elements, the influence of land use entropy, park density and street greenery are significant. Then, perceived environmental attributes influence SCB mainly through affecting neighborly interactions and community satisfaction. We also identify gender differences in the influence of neighborhood environment upon SCB and specifically males received more environmental restraints in terms of improving SCB. Given increasing awareness of the connection between neighborhood environment and SCB, neighborhood environment may prove to be valuable assets to improve individuals' psychosocial constructs such as SCB.

We acknowledge the limitations. First, due to the preference bias and socioeconomic favoritism, the evaluation of the perceived neighborhood environment variables could be overestimated or underestimated. Second, the 1,000-meter circular buffer was applied to calculate the objective built environment. However, results could differ if different buffer sizes are used. Third, this study focuses on cities with a higher population density which could confine the generalizability of the model and findings.

## Data availability statement

The original contributions presented in the study are included in the article/supplementary material, further inquiries can be directed to the corresponding authors.

## Author contributions

YD: conceptualization, methodology, and original draft and editing. HJ: conceptualization, supervision, and writing—review and editing. ZH: methodology, results analysis, and writing—review and editing. HY: supervision and writing—review and editing. All authors contributed to the article and approved the submitted version.
